# Visual Performance of Tecnis ZM900 Diffractive Multifocal IOL after 2500 Implants: A 3-Year Followup

**DOI:** 10.1155/2010/717591

**Published:** 2010-10-05

**Authors:** Leonardo Akaishi, Rodrigo Vaz, Graziela Vilella, Rodrigo C. Garcez, Patrick F. Tzelikis

**Affiliations:** Brasilia Ophthalmologic Hospital (HOB), SQSW 306, Bloco A, Apartment 105, Brasilia DF 70640-000, Brazil

## Abstract

*Purpose*. To evaluate visual performance for near, intermediate, and distant vision; complaints of photic phenomena, and patient satisfaction with the new diffractive multifocal IOL used in eyes which underwent phacoemulsification. *Methods*. Two thousand and five hundred consecutive eyes undergoing Tecnis ZM900 multifocal IOL implantation were included in this retrospective analysis. The minimum followup of 3 months was required after the surgery. Patients were assessed for uncorrected near visual acuity (UNVA) at a fixed distance (33 cm), uncorrected intermediate visual acuity (UIVA) at 60 cm, and uncorrected distance visual acuity (UDVA). Using a subjective questionnaire, patients satisfaction, their independence from using glasses, and the perception of glare and halo phenomena were also evaluated at the last follow-up. *Results*. Two thousand and five hundred eyes of 1558 patients underwent cataract surgery and Tecnis ZM900 multifocal IOL implantation. Four hundred and eighty seven patients (31.3%) were men, and 1071 (68.7%) were women. The mean age of the patients was 66.17 years. A UDVA of 20/30 or better was achieved by 85% of eyes. A UNVA of J1 was achieved by 93.7% of eyes and that of J2 or better was achieved by 98%. A UIVA of J4 or better was achieved by 65% and J5 or better was achived by more than 82.8% of the eyes in the study. Glare and halos were reported as severe by only 6.1% and 2.12% of patients, respectively. Ninety seven percent reported complete spectacle independence and 88% stated that they are totally satisfied with their quality of vision and would choose to have the same lens implanted again after the first implant. Five percent of the eyes in the study needed a second procedure (enhancement) to achieve a better visual result. No patient underwent lens exchange. *Conclusion*. Excellent near, intermediate, and distant vision was observed in patients implanted with the Tecnis ZM900 diffractive multifocal IOL. Spectacle independence and a minimum occurrence of photic phenomena make this IOL an excellent option in patients with cataract.

## 1. Introduction

Advances in both IOL and phacoemulsification technology have enabled surgery to evolve from a procedure concerned with the safe removal of the cataract to a much more refined procedure to achieve the best possible postoperative refractive result. Management of presbyopia is a challenge for refractive surgeons. The standard intraocular lens (IOL) implanted after cataract extraction to replace the focusing power of the natural lens has a single, fixed, focal length (monofocal IOL). Multifocal IOLs have been designed with the intention of providing good unaided distant, intermediate, and near vision [1–5]. Along with this, the development of aspherical IOLs has meant an incremental increase in visual quality for patients who undergo multifocal IOL implants [6–9].

Multifocal IOLs address the principle of simultaneous vision. Incoming light is divided between 2 lens powers: one for distance vision and one for near vision [4]. Clinically, multifocal IOLs have been reported to provide patients with functional near and distant vision with acceptable satisfaction. Reduced image contrast and unwanted visual phenomena, including glare and halos, have also been associated with multifocal IOL performance [4–6].

The Tecnis (AMO - Model ZM900) multifocal IOL is a second-generation silicone diffractive 3-piece lens. The innovative aspect of the Tecnis multifocal IOL is an anterior modified prolate surface, which neutralizes the negative impact of spherical aberrations on function vision and a posterior full diffractive multifocal surface. The addition power is +4.0 at lens plane. We present a large single-site series of patients who had a Tecnis ZM900 multifocal IOL implanted after cataract surgery.

The purpose of this study was to assess distance, intermediate, and near visual performance in patients who had cataract surgery with Tecnis ZM900 multifocal IOL implantation.

## 2. Patients and Methods

This retrospective study included patients with cataract, no indication of existing ocular pathology, unsatisfactory correction with glasses, visual potential in operative eye of 20/25 or better, and less than 1.50 diopters (D) of topography cylinder. Patients were offered the opportunity to be part of a clinical trial in which they would be allocated to have cataract surgery with a Tecnis multifocal IOL implant. Written informed consent was obtained from all patients before surgery, and the study was approved by the local ethics committee. Exclusion criteria were history of ocular trauma or prior ocular surgery, glaucoma or intraocular pressure greater than 21 mmHg, amblyopic eyes, retinal abnormalities, diabetes mellitus, steroid or immunosuppressive treatment, corneal and pupil abnormalities, capsule or zonular abnormalities, and connective tissue diseases. The selected lens used in this study was the Tecnis multifocal IOL (Model ZM900, AMO): a silicon foldable 3-piece IOL with 6.00 mm biconvex optic. 

Preoperative visit included an assessment of subject qualifications for inclusion in the study according to the protocol inclusion/exclusion criteria. Key data collection was medical history, uncorrected distance visual acuity (UDVA), best corrected distance visual acuity (CDVA), spherical equivalent (SE), and a subject lifestyle questionnaire. All patients had a complete ocular examination including refraction, intraocular pressure, and slit-lamp and fundus examination with dilated pupil. Preoperative testing included axial length measurement by partial coherence interferometry (IOL Master; Carl Zeiss Meditec, Jena, Germany). Intraocular lenses were calculated for a final refraction of 0 (emmetropia). Data recorded from the surgical procedure included lens serial number, lens power, and complications. 

The postoperative evaluation included uncorrected distance visual acuity (UDVA), uncorrected intermediate visual acuity (UIVA), uncorrected near visual acuity (UNVA), best corrected distance visual acuity (CDVA); and spherical equivalent (SE). Near and intermediate visual acuities were measured using the Rosenbaum near acuity card (Richmond Products, Inc.) held at distances of 33 cm and 60 cm, respectively. Clinical data was collected preoperatively and 1, 3, 6, 12, 24, and 36 months postoperatively for each eye.

At the last followup, patients satisfaction, their independence from using glasses, and the perception of photic phenomena were assessed by a subjective questionnaire developed by the author. The subjects were specifically queried about glare (trouble seeing street signs due to bright light or oncoming headlights) and halos (rings around lights). Patients rated the effect of each phenomenon on a scale from 0 to 3, with 0 meaning not observed, 1 as easily tolerated (interpreted as mild), 2 being defined as moderate, and 3 being defined as severe. 

All patients were operated using the same technique. All patients were topically anesthetised by lidocaine 2% gel before surgery. A 2.75 mm self-sealing clear cornea incision was made on the temporal side. Sodium hyaluronate 3%—chondroitin sulfate 4% (Viscoat) was used to reform and stabilize the surgical planes and protect the endothelium. A 5.00 to 5.25 mm continuous curvilinear capsulorhexis was initially performed with a 26-gauge needle and completed with forceps. Phacoemulsification was performed using the Infinity machine (Alcon Surgical) or Sovereign machine (Allergan Surgical). All IOLs were planned to be inserted in the capsular bag with the injector system. The viscoelastic material was completely removed at the end of the procedure. No sutures were used in any case. In patients with corneal astigmatism, between 0.75 D and 1,50 D, verified by topography, limbal relaxing incisions were performed according to Gill's modified nomogram. Postoperative medication included moxifloxacin (Vigamox) or gatifloxacin (Zymar) 4 times a day for 2 weeks, 0.1% diclofenaco sodium (Voltaren) 3 times a day for 4 weeks, and steroid (Predfort) eyedrops 4 times a day for 6 weeks. The minimum postoperative followup for inclusion in the study was 3 months.

 The enhancement rate was also evaluated, that is, the quantity of cases in which a second procedure was needed to achieve the desired refractive result. The types of procedures used in enhancement were also described.

All data analysis was performed using SPSS statistical software package for Windows (version 17.0 SPSS Inc. Chicago, IL). For statistical analysis of visual acuity, logarithms of minimum angle of resolution (logMAR) acuity values were used. Descriptive statistics (mean, minimum, maximum, SD) were calculated for age, spherical equivalent, refraction, and visual acuity. The paired-sample *t*-test was used to compare preoperative and postoperative spherical equivalents. A *P* value less than.05 was considered statistically significant.

## 3. Results

Two thousand and five hundred eyes of 1558 patients were included in the study. One thousand and seventy one (68.7%) were women, and 487 (31.3%) were men. Nine hundred and forty two patients (60.5%) received bilateral implants while 616 patients (39.5%) received unilateral implants. The mean age of the patients was 66.17 years ± 8.94 (SD) (range 34 to 87 years) (Table 1). Patients were followed up for an average of 13.6 months (range 3 to 36 months). In 1042 eyes (41.68%), associated limbal relaxing incisions were made.

**Figure 1:**
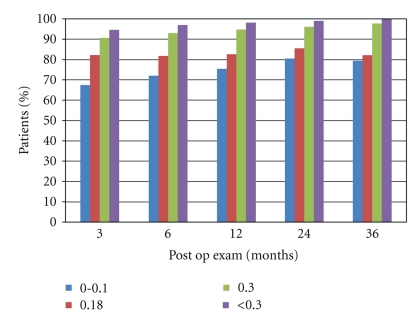
UDVA in different periods of followup.

Preoperatively, the mean logMAR UDVA of these eyes was 0.61 ± 0.35 (range 0 to 1.3). After a followup of 36 months postoperatively, the mean UDVA was 0.06 ± 0.09 (range 0 to 0.54). The UDVA was significantly better at all followup periods compared to that before referral (*P* < .05). In Table 2, shows the UDVA in different periods during the followup. In Figure 1, it can be observed that an UDVA of 0.10 or better was achieved by 67.64% of the eyes in all periods of followup. It was also found that, no matter what followup period was observed, a UDVA of 0.18 or better was achieved in approximately 85% of the eyes. Finally, it was also observed in all followup periods that an UDVA of 0.30 or better was achieved in approximately 95% of the eyes.

Preoperatively, the mean logMAR CDVA of these eyes was 0.13 ± 0.17 (range 0 to 1.3); After the surgery, with an average followup, the mean logMAR CDVA of patients was 0.01 ± 0.03 (range 0 to 0.3), this was significantly better than before referral (*P* < .05); Table 3.

**Figure 2:**
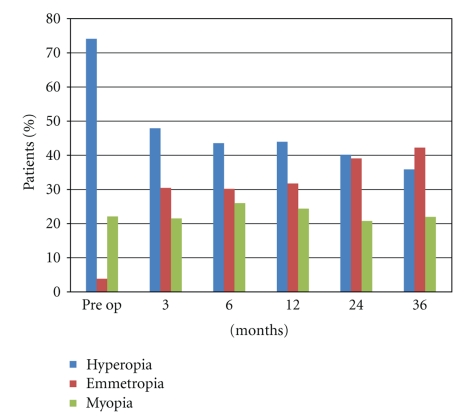
Spherical equivalent in pre-op and in different periods of followup.

The average SE at referral of these eyes was +1.02 D (range −12.50 to +12.25 D). Five hundred and fifty five eyes (22.2%) had myopic spherical equivalent, the mean was −2.07 (range −12.50 to −0.25 D). Ninety seven eyes (3.88%) emmetrope SE and, finally, 1848 eyes (73.92%) had hyperopic spherical equivalent, the mean was +2.01 (range +0.25 to +12.25 D). Postoperatively, the average SE was +0.08 D (range −0.75 to +1.25 D). Table 4 and Figure 2 show the SE in different periods during the followup.

**Figure 3:**
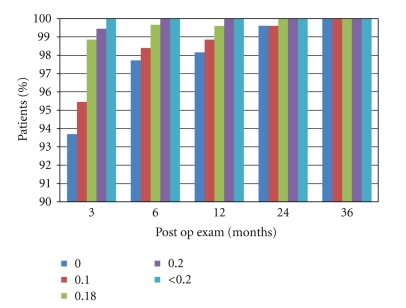
Post-op UNVA in different periods of followup.

Table 5 shows the uncorrected near visual acuity (UNVA) performance of these eyes in each postoperative period. The mean logMAR UNVA in all the postoperatory periods was 0.00. In all followup periods, the UNVA was found to be better than 0.00 in 93.7% and 0.1 or better in 98% of the eyes in study (Figure 3).

**Figure 4:**
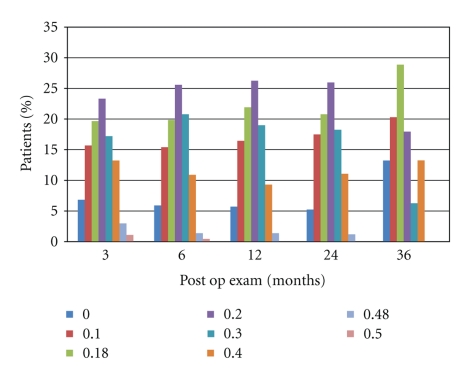
Post-op UIVA in different periods of followup.

Table 6 shows the uncorrected intermediate visual acuity (UIVA) performance of these eyes in each post operative period. The mean logMAR UIVA in all the postoperatory periods was 0.18. In all followup periods it was found 0.18 or better in 41% of the eyes; 0.20 or better in more than 65% of the eyes 0.30 or better in more than 82.8% of the eyes in study (Figure 4).

 A qualitative performance analysis of the IOL, by means of a subjective questionnaire, took into consideration only results of patients with bilateral implants. At the last followup, 97.87% (922 of 942 patients) of the patients were spectacle independent for near and distant vision and 88% (829 of 942 patients) of the patients were totally satisfied with their quality of vision. After an average followup of 13.6 months, for glare after the second implant, of the 942 patients who answered the question 6.1% (*n* = 58) rated their observation as severe in effect, 26.2% (*n* = 247) rated it as moderate, and 67.7% (*n* = 637) rated it as none or mild. Halos were reported as severe by 2.12% of patients (*n* = 20), moderate by 16.45% (*n* = 155), and absent or mild by 81.43% (*n* = 767). No other complaints were reported.

With regard to enhancement, 5.24% (131 out of 2500 eyes) needed to undergo a second procedure in order to achieve the desired refraction result. 57 eyes (2.28%) were submitted to excimer laser correction, while 74 eyes (2.96%) had the optical zone of the IOL placed over the capsulorhexis (buttoning of the optical zone in the capsulorhexis for slight residual hyperopia correction). The following was also undertaken: Pars Plana vitrectomy in 26 eyes (1.04%); YAG laser in 88 eyes (3.52%). There were retina detachment in 1 eye, cystoid macular edema in 5 eyes, and rupture of the posterior capsule in 14 eyes. In 3 of the 14 eyes with rupture of the posterior capsule, the IOL was fixed on the iris. No implant replacement was done.

## 4. Discussion

The loss of accommodation following cataract surgery and the restoration of near vision in patients with IOL implantation remains a challenging problem of modern cataract surgery. Because the natural process of accommodation is not restored, a monofocal IOL needs complementary reading or multifocal glasses to create good vision at more than one distance. Recently, multifocal IOLs have been investigated and gained wide popularity. 

The first multifocal IOL approved for general use in the United States is the Array (AMO, Advanced Medical Optics, Santa Ana, California) [10]. The Array is a zonal progressive intraocular lens with five concentric zones on the anterior surface. Zones 1, 3, and 5 are distance dominant zones whereas zones 2 and 4 are near dominant. The lens has an aspheric design, and each zone repeats the entire refractive sequence, corresponding to distant, intermediate, and near foci. A study that evaluated the clinical outcomes and patient satisfaction after implantation of multifocal IOLs found that multifocal IOLs are more effective at improving uncorrected near vision acuity (UNVA) and have a reduced spectacle dependence for near and distant vision relative to monofocal IOLs [11]. 

The development of the prolate aspherical monofocal Tecnis IOL was a major step toward the reduction of ocular spherical aberration, resulting in improved visual function, particularly contrast vision. It was therefore a logical step to incorporate the aspherical IOL platform into the design of a new multifocal IOL to counteract the negative impact of multifocal IOL design on contrast vision [12]. Based on the favorable optical performance of a diffractive PMMA bifocal IOL (811E, Pharmacia) [13], a diffractive optic design was then applied to the multifocal Tecnis IOL, with the result being the Tecnis ZM900. The Tecnis (AMO - Model ZM900) multifocal IOL is a second-generation silicone diffractive 3-piece lens. The innovative aspect of the Tecnis multifocal IOL is an anterior modified prolate surface, which neutralizes the negative impact of spherical aberrations on function vision, and a posterior full diffractive multifocal surface with the addition power of +4.0 at lens plane. The present study presents a large number of surgeries with a long longitudinal followup.

The diffractive IOLs use diffraction and interference to form multiple discrete foci. These IOLs can be considered conventional monofocal lenses with the diffractive element on one surface. The effect of the diffractive element is to split most of the incident light into the zeroth (distance) and first (near) diffracted orders. Of the incident light, approximately 41% goes to the near foci, 41% to the distance foci, and the remaining light goes into higher orders. The full-aperture diffractive maintains the ratio for all pupil sizes. A theoretical study on model eyes showed that diffractive multifocal IOLs are superior to refractive multifocal IOLs for near vision whereas for distant vision they are comparable [14]. 

Although the Tecnis multifocal IOL made of acrylic is presently available, this study brings together results of implants with the IOL made of silicone. The reason being that, whereas the silicone IOL has been used since January 2006, the acrylic IOL only came on the market in June 2008. We believe that the availability of this new material will make a further contribution to the improved quality of vision, reducing modulation transfer function (MTF) through the correction of chromatic aberration. We have already begun a new study to evaluate the performance of the acrylic IOL. 

In order to analyze IOL quantitative performance, results of patients who received unilateral implants as well as patients who got Tecnis multifocal lenses in both eyes were studied. That is because evaluations of visual acuity and refraction in each eye were individualized. In other words, the analysis was made of the contralateral occluded eye. As for the IOL qualitative analysis made through a subjective questionnaire, only the results of patients who underwent bilateral implants were used. The reason for this is that the observations were made by the patient during day-to-day activities, that is, with both eyes open; a different condition from contralateral eye (e.g., phakic eye, with monofocal IOL implant or with a multifocal IOL implant that is not Tecnis) would alter patient observations compromising results (positive or negative) related exclusively of the Tecnis IOL.

Numerous studies have already shown the excellent quantitative and qualitative performance of Tecnis multifocal IOLs [5, 6, 9]. Cillino et al. [15]. found that the new-generation multifocal IOLs (Tecnis ZM900) provided better near vision, equivalent intermediate vision, less unwanted photic phenomena, and greater spectacle independence than either monofocal (AR40, AMO) or refractive multifocal IOLs (ReZoom, AMO; Array, AMO). Contrary to findings of Cillino et al., a study by Palmer et al. [16] showed a better visual function and lesser photic phenomena with monofocal IOLs (Tecnis Z900, AMO) compared to multifocal IOLs (Tecnis ZM900, AMO; ReZoom, AMO; TwinSet, Acri.Tec) but patients were spectacle dependent. Artigas et al. [17], also analyzed the image quality (MTF) of 4 IOLs (ReSTOR, Alcon; Tecnis ZM900, AMO; ReZoom, AMO; SN60WF, Alcon) and observed that the reference monofocal IOL provided better distance images than any multifocal IOL with all pupil sizes. In a retrospective study by Ngo et al. [18], 108 eyes of 54 patients received 1 of 3 IOLs (Tecnis ZM900, SA60D3, SN60D3). All three IOLs provide similar uncorrected and best corrected distance visual acuity. However, the ZM900 IOL provided better binocular distance corrected and uncorrected near acuity than the 2 other lenses.

In the present study, the mean UDVA reached excellent levels in all the followups. Other studies describing Tecnis ZM900 multifocal IOL also achieved similar results. In a prospective study by Cillino et al. [15], eyes that received the Tecnis ZM900 IOL achieved a mean logMAR UDVA of 0.18 after 1 year of followup. Ngo et al. [18] also found a mean logMAR UDVA of 0.10 after 3 months of followup, with an UDVA of 20/20 or better in 30.0% of the eyes implanted with Tecnis ZM900, an UDVA of 20/30 or better in 86.6%, and an UDVA of 20/40 or better in 96.7% of the eyes. Santhiago and associates [19] conducted a prospective study in which 40 eyes of 20 patients received one of two multifocal IOLs (Tecnis ZM900, and ReSTOR SN60D3). After 3 months of followup the mean logMAR UDVA was 0.12 and 0.14 in the Tecnis ZM900 and ReSTOR group, respectively. 

In all followups, the mean UIVA and UNVA reached clinically useful values, better than 20/30 (0.18) in 41% and better than 20/25 (0.10) in 98% of the eyes, respectively. In a study by Cillino and associates, they also found that Tecnis ZM900 IOL provided a significant improvement in UIVA and UNVA, with a mean of 20/23 and 20/25, respectively. Palmer et al. conducted a prospective study in which 114 patients (228 eyes) received one of four IOLs (Tecnis Z9000, Tecnis ZM900, ReZoom, Acri.Tec) [16]. At the last followup, patients implanted with the Tecnis ZM900 showed significant better UNVA compared with others IOLs. In another study of Hütz et al. [9], they retrospectively evaluated reading performance at intermediate distances of three types of multifocal IOLs (Tecnis ZM001, Array, ReSTOR) under different light conditions based on reading acuity and reading speed tests and the Tecnis IOL provided better uncorrected reading speed at intermediate distances than array and ReSTOR IOLs. 

In our study, the use of limbal relaxing incisions (LRIs) during implantation of the Tecnis ZM900 multifocal IOL was done in 41.68% of the surgeries. Estimates of the incidence of naturally occurring astigmatism vary; it has been reported that at time of cataract surgery in a general population, the cylinder is more than 2.00 diopters (*D*) in approximately 10% of patients, between 1.00 D and 2.00 D in 20% and less than 1.00 D in 70% [20]. Although it is possible to reduce astigmatism with LRIs, further correction with LASIK may be needed for larger amounts of residual spherical and cylindrical errors [21]. Also, because multifocal IOLs split the light, patients with these IOLs may be more sensitive to changes caused by residual astigmatism. Other studies of multifocal IOL implantation combined with LRIs were also effective and safe in reducing pre-existing corneal astigmatism [21, 22]. 

Despite advances in IOL calculation, there may be a residual refractive error after multifocal IOL implantation that necessitates correction with spectacles or another surgery to provide satisfactory distance and near acuity [23]. The best strategy for a second procedure (enhancement) should be decided upon by taking into account not only the situation to be corrected, but eye conditions as well. A new refractive surgery, a secondary additional implant and even a change of IOL, are some examples. Five percent of the eyes (*n* = 131) needed to undergo a second procedure in order to achieve the desired refraction result. Fifty seven eyes (2.28%) were submitted to excimer laser correction. Studies [24, 25] also report good results for the correction of refractive errors with LASIK or photorefractive keratectomy (PRK) after multifocal IOL implantation. Alfonso et al. [26] found good outcomes in 53 eyes of 31 patients that had LASIK for residual refractive error after refractive lens exchange (RLE) with implantation of an AcrySof ReSTOR IOL. Recently, Muftuoglu et al. [27] also reported that LASIK after RLE with AcrySof ReSTOR IOL implantation was effective in 85 eyes of 59 patients. 

In this study, 74 eyes (2.96%) had the IOL optic captured through the continuous curvilinear capsulorhexis (CCC) anteriorly to correct a residual refractive error. A change occurs in the position of the IOL optical zone: moving it to a forward position, so the optical zone buckles on the anterior capsulorrexis (for the correction of small hyperopic errors), or moving it backward, so the optical zone buckles on a posterior capsulorrexis that is created (for correction of small myopic errors). These are viable alternatives in cases where a new corneal procedure is impossible. We first describe this technique of anterior optic capture of a multifocal IOL to correct residual refractive error in 2009 [27].

The results show the improvement that can be achieved after implantation of these lenses, which have characteristics that can correct distant, intermediate, and near vision after cataract surgery. We believe that IOL selection should be based on patient preference and daily activities. Overall, the use of a Tecnis ZM900 multifocal IOL appears to be a safe and efficient procedure and a good refractive solution. Attention to detail in regard to proper patient selection, preoperative measurements, intraoperative technique, and postoperative management has resulted in excellent outcomes and improved patient acceptance of this effective technique. As with all refractive procedures, realistic expectations should be established prior to surgical intervention.

## Figures and Tables

**Table 1:** Preoperative characteristics of the patients (*N* = 1558).

Characteristics	Value
Sex, *n* (%)	
Male	487 (31.3)
Female	1071 (68.7)
Age (y)	
Mean ± SD	66.17 ± 8.94
Range	34 – 87
Mean CDVA (logMAR) ± SD	0.13 ± 0.17
Mean SE (D) ± SD	+1.02 ± 2.22

CDVA = corrected distance visual acuity; SE = spherical equivalent.

**Table 2:** UDVA in different periods during the followup.

	PRE-	Postoperatory				
	Pre-op	3° mth	6° mth	12° mth	24° mth	36° mth
0.00	57 (2.28%)	1059 (42.36%)	1193 (47.77%)	1199 (48.34%)	433 (56.52%)	74 (57.81%)
0.10	117 (4.68%)	632 (25.28%)	606 (24.26%)	675 (27.21%)	185 (24.15%)	28 (21.87%)
0.18	162 (6.48%)	441 (17.64%)	456 (18.26%)	435 (17.54%)	111 (14.49%)	23 (17.96%)
0.30	344 (13.76%)	234 (9.36%)	171 (6.84%)	128 (5.16%)	28 (3.65%)	3 (2.34%)
0.40	194 (7.76%)	77 (3.08%)	34 (1.36%)	31 (1.25%)	3 (0.39%)	—
0.48	231 (9.24%)	37 (1.48%)	20 (0.8%)	6 (0.24%)	3 (0.39%)	—
0.54	259 (10.36%)	14 (0.56%)	14 (0.56%)	6 (0.24%)	3 (0.39%)	—
0.60	145 (5.8%)	3 (0.12%)	3 (0.12%)	—	—	—
0.70	202 (8.08%)	3 (0.12%)	—	—	—	—
0.88	316 (12.64%)	—	—	—	—	—
1.00	202 (8.08%)	—	—	—	—	—
1.18	20 (0.8%)	—	—	—	—	—
1.30	251 (10.04%)	—	—	—	—	—
Total	2500	2500	2497	2480	766	128
	100%	100%	100%	100%	100%	100%

**Table 3:** CDVA in different periods during followup.

	PRE-	Postoperatory				
	Pre-op	3° mth	6° mth	12° mth	24° mth	36° mth
0.00	1082 (43.28%)	2048 (81.9%)	2079 (83.25%)	2075 (83.67%)	702 (91.65%)	116 (90.63%)
0.10	524 (20.96%)	361 (14.46%)	365 (14.61%)	359 (14.48%)	54 (7.05%)	9 (7.04%)
0.18	342 (13,68%)	74 (2.96%)	46 (1.84%)	42 (1.69%)	6 (0.78%)	2 (1.55%)
0.30	328 (13.12%)	12 (0.46%)	5 (0.21%)	3 (0.11%)	4 (0.52%)	1 (0.78%)
0.40	99 (3.96%)	5 (0.22%)	2 (0.09%)	1 (0.05%)	—	—
0.48	41 (1.64%)	—	—	—	—	—
0.54	18 (0.72%)	—	—	—	—	—
0.60	18 (0.72%)	—	—	—	—	—
0.70	20 (0.80%)	—	—	—	—	—
0.88	8 (0.32%)	—	—	—	—	—
1.00	18 (0.72%)	—	—	—	—	—
1.18	—	—	—	—	—	—
1.30	2 (0.08%)	—	—	—	—	—
Total	2500	2500	2497	2480	766	128
	100%	100%	100%	100%	100%	100%

**Table 4:** Spherical equivalent in different periods during followup.

	PREOP	3 months	6 months	12 months	24 months	36 months
SE Myopia						
*N*	555	541	652	603	159	28
% of patients	22.2	21.64	26.11	24.31	20.75	21.87
Mean (D)	−2.7	−0.42	−0.46	−0.44	−0.40	−0.37
SE Emmetropia						
*N*	97	760	755	786	299	54
% of patients	3.88	30.40	30.23	31.69	39.03	42.18
SE Hyperopia						
*N*	1848	1199	1090	1090	308	46
% of patients	73.92	47.96	43.65	43.95	40.20	35.93
Mean (D)	+2.01	+0.51	+0.46	+0.43	+0.42	+0.43

SE: spherical equivalent.

**Table 5:** UNVA in each postoperative period.

	3° mth	6° mth	12° mth	24° mth	36° mth
0.00	2343 (93.72%)	2440 (97.71%)	2434 (98.14%)	763 (99.6%)	128 (100%)
0.10	114 (4.56%)	40 (1.6%)	29 (1.16%)	3 (0.4%)	—
0.18	29 (1.16%)	9 (0.36%)	14 (0.56%)	—	—
0.20	14 (0.56%)	—	3 (0.12%)	—	—
< 0.20	—	8 (0.32%)	—	—	—
Total	2500	2497	2480	766	128
	100%	100%	100%	100%	100%

**Table 6:** UIVA in each postoperative period.

	3° mth	6° mth	12° mth	24° mth	36° mth
0.00	171 (6.84%)	148 (5.92%)	142 (5.72%)	40 (5.22%)	17 (13.28%)
0.10	393 (15.72%)	384 (15.37%)	407 (16.41%)	134 (17.49%)	26 (20.31%)
0.18	492 (19.68%)	495 (19.82%)	544 (21.93%)	159 (20.75%)	37 (28.9%)
0.20	584 (23.36%)	638 (25.55%)	652 (26.29%)	199 (25.97%)	23 (17.96%)
0.30	430 (17.2%)	518 (20.74%)	470 (18.95%)	140 (18.27%)	8 (6.25%)
0.40	330 (13.2%)	271 (10.85%)	231 (9.31%)	85 (11.09%)	17 (13.28%)
0.48	74 (2.96%)	34 (1.36%)	34 (1.37%)	9 (1.17%)	—
0.50	26 (1.04%)	9 (0.36%)	—	—	—
Mean UCIVA	J 3.85	J 3.79	J 3.69	J 3.72	J 2.47
Total	2500	2497	2480	766	128
	100%	100%	100%	100%	100%
